# Social Integration, Social Support and Incident Type 2 Diabetes: Results from the Prospective Heinz Nixdorf Recall Cohort Study

**DOI:** 10.3390/epidemiologia7040106

**Published:** 2026-08-04

**Authors:** Bernd Kowall, Nico Dragano, Börge Schmidt, Andreas Stang

**Affiliations:** 1 Institute of Medical Informatics, Biometry and Epidemiology, University Hospital Essen, University Duisburg-Essen, 45147 Essen, Germany; 2 Institute of Medical Sociology, Centre for Health and Society, Medical Faculty and University Hospital, University of Düsseldorf, 40225 Düsseldorf, Germany

**Keywords:** diabetes, socioeconomic status, social support, social integration, isolation, RERI

## Abstract

*Background and Objectives*: Aspects of social relationships, like isolation and low social support, have been linked to type 2 diabetes onset, though evidence is inconsistent. We investigated whether social relationships mediate the association between socioeconomic status (SES) and diabetes or interact with SES in relation to diabetes. *Materials and Methods:* The study included 3642 participants (mean age 58.9 ± 7.6 years, 47.9% male) from the Heinz Nixdorf Recall Study, a population-based cohort in Germany. Baseline examinations took place in 2000–2003 with two 5-year follow-ups. Adjusted relative risks (RRs) were estimated for social relationships (isolation, partnership, instrumental, financial, emotional support) and incident diabetes using Poisson regression with robust variance. We performed causal mediation analyses and estimated relative excess risks due to interaction (RERI). *Results*: Associations between measures of social integration and diabetes incidence were weak (e.g., RR = 1.09 (95% CI: 0.86, 1.39) for lack of partnership). Limited financial support was associated with higher diabetes risk (RR = 1.33 (1.09, 1.62)), whereas instrumental or emotional support was not. Some evidence of mediation was found for limited financial support (14% (95% CI: −7%, 35%) for the income–diabetes association). There was hardly any excess risk of diabetes in persons with poor education, and poor income, respectively, and low social support (RERI = −0.33 to 0.27). *Conclusions*: In this prospective study, limited financial support emerged as a social risk factor for type 2 diabetes and explained a small proportion of socioeconomic inequalities in diabetes incidence. Other dimensions of social relationships showed little evidence of independent, mediating, or interaction effects. The findings regarding financial support require confirmation in further studies.

## 1. Introduction

Social relationships influence health, as demonstrated by the increased mortality risk observed in socially isolated individuals [1,2]. Accordingly, the World Health Organization (WHO) recently reported that loneliness and social isolation have a substantial impact on health and well-being, identifying them as major public health challenges [3].

However, there is little research on the impact of different aspects of social relations on incident type 2 diabetes. Moreover, most diabetes-related studies have focused on quantitative dimensions of social relationships, such as the number and strength of ties, while quality (e.g., social support) is seldom studied.

A recent meta-analysis including cohort and case-control studies suggests that social isolation is a risk factor for diabetes leading to a risk increase of about 20% [4]. Similarly, loneliness—which can be experienced even in the presence of others—has been associated with a 32% increased risk [4]. Larger pooled effects were observed in another meta-analysis, which also included cross-sectional studies [5]. Proposed mechanisms include stress and depression acting as mediators between social isolation and incident diabetes [6]. Moreover, social isolation may also affect behaviors, such as reduced physical activity, and, thus, lead to an increased risk of diabetes [7,8]. Further studies on the association between aspects of social relations and incident diabetes yielded inconsistent results, with some indicating sex differences [9,10,11,12]. Some studies suggest that being married is associated with a lower risk of developing diabetes [12,13]. Furthermore, few studies suggest that persons with lower levels of social support are at a higher risk of diabetes [10,11,14,15].

It is well established that individuals with a lower socioeconomic status (SES) are at an increased risk for developing diabetes [16]. A scoping review reported inconsistent findings on whether social network variables mediate the relationship between SES and health outcomes, as well as on whether social network variables interact with SES concerning health outcomes [17]. In particular, for diabetes, it is unclear whether social integration and social support act as mediators in the pathway between SES and incident diabetes, or whether individuals with low SES, who also have limited social networks or social support, face a diabetes risk that synergistically exceeds the sum of risks from the individual factors.

The first aim of the present study was to examine the association between social integration and different forms of social support on incident diabetes in a large, population-based cohort study. The second aim was to explore the potential mediating effects of social integration and social support on the association between socioeconomic status and the risk of developing diabetes, as well as the potential interaction between characteristics of social relationships and SES with regard to incident diabetes.

## 2. Materials and Methods

### 2.1. Study Population

We used data from the Heinz Nixdorf Recall Study (HNRS), which is a population-based prospective cohort study conducted in three large adjacent cities (Bochum, Essen, Mülheim) in the Ruhr region of North-Rhine-Westphalia, Germany. The study rationale and design have been described in detail elsewhere [18]. In short, the cohort comprises a total of 4814 participants (49.8% men, aged 45–75 years). The baseline examination (T0) was conducted between 2000 and 2003. The median follow-up time was 5.1 years for the 5-year follow-up visits between 2006 and 2008 (T1), and 5.2 years for the 5-year follow-up visits between 2011 and 2015 (T2). Data assessment at baseline and at follow-up visits included a self-administered questionnaire, face-to-face interviews, and a physical examination including, among others, anthropometric measurements and comprehensive laboratory tests. The study was approved by the ethics committee of the University of Duisburg-Essen. All participants gave their written informed consent.

The analysis dataset comprised 3642 individuals who were free of diabetes at baseline and had at least one diabetes measurement at either T1 or T2. Of the 4814 participants at T0, 576 were excluded because they had diabetes at baseline and 47 due to missing diabetes measurement at T0, while a further 523 did not participate in T1 and T2, and a further 26 were excluded due to missing diabetes measurements at T1 and T2.

### 2.2. Measurement and Definition of Study Variables

Information on education, marital status, partnership, health status, medication intake, income, social ties and social support were collected through interviews and questionnaires. HbA1c was measured using a nephelometer (BN-II, Dade-Behring Inc., Deerfield, IL, USA).

Persons were diagnosed with prevalent (T0) or incident type 2 diabetes (T1, T2) if at least one of the following criteria was fulfilled: self-report of diabetes, intake of antidiabetic drugs (ATC code A10) or insulin, HbA1c ≥ 6.5%. As the age rate at baseline was 45 to 75 years, we assumed that there were no incident cases of type 1 diabetes.

Educational attainment was assessed using the International Standard Classification of Education (ISCED-97) [19], which classifies individuals into seven categories. In the analyses, educational attainment was dichotomized, contrasting up to higher secondary to post-secondary education. Equivalent household income was calculated using information on disposable income and the number of adults and children in the household according to the “OECD-modified scale” [20]. In the analyses, individuals in the lowest household income quartile (Q1) were either compared to individuals in the three higher income quartiles (Q2–Q4) or to individuals in the highest quartile (Q4).

We used the social integration index (SII) developed by Berkman et al., which takes three components into account: partnership, close ties, and participation [1]. Participants received 2 points if they were married or had a steady partner, and 0 points otherwise. Close ties were defined as individuals reporting that they met their children, relatives, or friends at least once a month. The scoring for close ties was as follows: 0 points for 0–2 close ties, 1 point for 3–11 close ties, and 2 points for more than 11 close ties. Regarding participation, participants were presented with a list of nine clubs and organizations. Based on their involvement, they received 0 points for no participation, 1 point for participation in one, and 2 points for participation in two or more organizations. The SII ranged from 0 to 6 points and was categorized into four levels: Level I (0–1 point, social isolation), Level II (2–3 points), Level III (4–5 points), and Level IV (6 points). In regression analyses, the SII was dichotomized by contrasting the combined levels I and II to the combined levels III and IV.

Three components of social support provided by partners, relatives, friends, neighbors, colleagues and others were assessed: instrumental support, financial support and emotional support. For each component, the degree of support was classified into four categories: available and sufficient; available but not needed; available but insufficient; and not available.

### 2.3. Statistical Analysis

Baseline characteristics of the study sample are presented as numbers (percentages) or as means with standard deviations. Complete case log-linear models with a Poisson working likelihood and robust standard errors were fitted to estimate crude and adjusted relative risks (RRs) for the association between exposure variables (household income, educational attainment, SII, partnership, functional support) and incident type 2 diabetes [21]. In the regression analyses and when estimating relative excess risks due to interaction (RERI), the measures of social support were dichotomized by combining the two latter categories (“available but insufficient” and “not available”) and contrasting them with the first two categories (“available and sufficient” and “available but not needed”). We used Directed Acyclic Graphs (DAGs) to find minimal adjustment sets. For models with household income or educational attainment as exposure variables, the adjustment set included age and sex. For SII, and for financial, instrumental and emotional support, the adjustment set included age, sex, household income and educational attainment. We did not adjust for potential mediators between social integration and social support, respectively, and diabetes, such as lifestyle factors, sleep disorders, stress, depression, and BMI [14]. As household income, SII and partnership had also been measured at T1, we additionally fitted the same regression models for these variables with time-dependent exposures by defining the exposure separately for the time intervals T0 to T1 and T1 to T2 for each person.

For mediation analysis, the SAS (version 9.4; SAS Institute, Cary, NC, USA) procedure PROC CAUSALMED was used. The percentage mediated was calculated by dividing the indirect effect by the total effect. Exposure variables were household income (Q1 versus Q2–Q4), and educational attainment (up to higher secondary versus post-secondary). The outcome was incident diabetes, and age and sex were included as covariates. SII and the three types of functional support were included as potential mediators.

To assess whether individuals with both low SES and poor social support (defined as a lack of social contacts or lack of functional support) had a higher risk of diabetes than expected from the sum of the individual effects, we calculated relative excess risk due to interaction (RERI) (also called interaction contrast ratio) using the following formula [22,23]:

RERI = RR11 − RR10 − RR01 + 1.

Here, RR11 refers to the risk among individuals exposed to both risk factors, while RR10 and RR01 refer to risks in individuals exposed to only one of the two. A publicly available Excel sheet was used to calculate 95% confidence intervals for the RERIs [23]. A RERI of 0 indicates no interaction on the additive scale. For example, a RERI of 0.15 indicates that there is synergism, and the excess risk due to interaction is 15% of the risk among the double-unexposed.

To account for whether incident diabetes occurred between T0 and T1 or between T1 and T2, we additionally fitted accelerated failure time (AFT) models for interval-censored data. In these models, the expected time to diabetes among exposed individuals is exp(β) times the expected time among unexposed individuals. Compared to Cox proportional hazards models, AFT models have the advantage that the effect estimate retains the time dimension and can accommodate event time intervals when the exact time of onset is unknown. AFT models were also fitted separately for men and women.

In further additional analyses, we did not dichotomize the variables financial support, instrumental support, and emotional support but used all four exposure categories with “available but not needed” as the reference category. In the same way, we did an additional analysis taking all four categories of the SII into account.

## 3. Results

The analysis dataset included 3642 individuals (47.9% male; mean age 58.9 years) (Table 1). Educational attainment was classified as post-secondary (according to ISCED-97) for 1250 individuals (34.3%). Based on the SII, 6.8% of participants were categorized as socially isolated (Level I), while 40.1%, 47.7%, and 5.5% were classified as Levels II, III, and IV, respectively. Overall, 15.2% of participants reported having no partner. Financial support was either unavailable or insufficient for 20.9% of the sample. The corresponding proportions for insufficient instrumental and emotional support were 11.9% and 15.6%, respectively. The mean HbA1c level was 5.3%. Supplementary Table S1 shows baseline characteristics of excluded participants. Compared to included participants, excluded participants were about three years older and had a slightly lower household income. Moreover, social isolation at baseline was more prevalent among those who did not participate at follow-up any more.

Incident diabetes occurred in 449 individuals (12.3%). Table 2 presents the associations between socioeconomic and social factors and diabetes risk. Individuals in the lowest household income quartile had a higher age- and sex-adjusted risk of diabetes compared to those in the highest quartile (relative risk [RR] = 1.38; 95% confidence interval [CI]: 1.05–1.81). Participants with a lower educational level (up to higher secondary) also had an increased diabetes risk compared to those with post-secondary education (RR = 1.19; 95% CI: 0.98–1.43). The adjusted diabetes risk of individuals with low SII (levels I or II) barely differed from the risk of people with more frequent contacts (level III or IV) (RR = 1.05 (95% CI: 0.87–1.26)). Comparing individuals without a partner to individuals with a partner, the adjusted RR was 1.09 (95% CI: 0.86–1.39). When people reporting unavailable or insufficient social support were compared to persons who either received sufficient support or did not require it, the adjusted RRs were 1.33 (95% CI: 1.09–1.62), 1.12 (95% CI: 0.86–1.45) and 0.89 (95% CI: 0.69–1.14) for financial, instrumental and emotional support, respectively.

In supplementary analyses with time-dependent exposure, the associations between household income and incident diabetes increased. The relative risk was 1.55 (95% CI: 1.18–2.04) for comparing the lowest to the highest household income quartile, and 1.30 (95% CI: 1.06–1.60) for comparing the lowest to the three higher household income quartiles. For social contacts and partnership, however, the relative risks changed only marginally (RR = 1.10 (95% CI: 0.91–1.34) for comparing SII levels I or II to levels III or IV, and RR = 1.12 (95% CI: 0.87–1.45) for no partnership versus having a partner.

In further supplementary analyses, we used all four categories of SII, financial support, instrumental support, and emotional support (Supplementary Table S2). Isolated persons have a diabetes risk that is only slightly larger than the risk of persons with a large network (RR = 1.10 (95% CI: 0.69–1.75)). For the exposure contrast “support not available” versus “support available, but not needed” (=reference) higher relative risks were seen in the adjusted models: RR = 1.41 (95% CI: 1.14–1.73) for financial support, RR = 1.72 (95% CI: 1.24–2.37) for instrumental support, and RR = 1.28 (95% CI: 0.87–1.90) for emotional support.

A total of 14.2% (95% CI: −6.7%, 35.0%) of the effect of household income on incident diabetes was attributed to the mediation of financial support, whereas financial support accounted for hardly any portion of the effect of education on incident diabetes (Table 3). For SII and instrumental support, the percentage mediated ranged from 2% to 5%. For emotional support, the percentage mediated was slightly negative (−3.0% and −0.8%, respectively, for household income and education).

A further additional analysis showed that persons with a household income below the median had a higher risk of low financial support (22.9% for income below the median, 18.8% for income above the median; risk difference = 4.1% (95% CI: 1.4%; 6.9%)). In the quartiles of household income, the proportion of those with available, but not needed financial support increased from 55.1% (lowest income) to 65.6%, 67.8% and 69.4%, respectively, for quartiles with higher income.

Analyses of the interaction between social integration and social support and education yielded small RERIs, which were all close to 0 (Table 4). For low education and low instrumental support, the excess risk was 23% (95% CI: −41%, 86%) of the risk of persons with high education and high instrumental support. When household income was used as the measure of socioeconomic status (Supplementary Table S3), RERIs were all close to 0 or smaller than 0.

**Figure 1:**
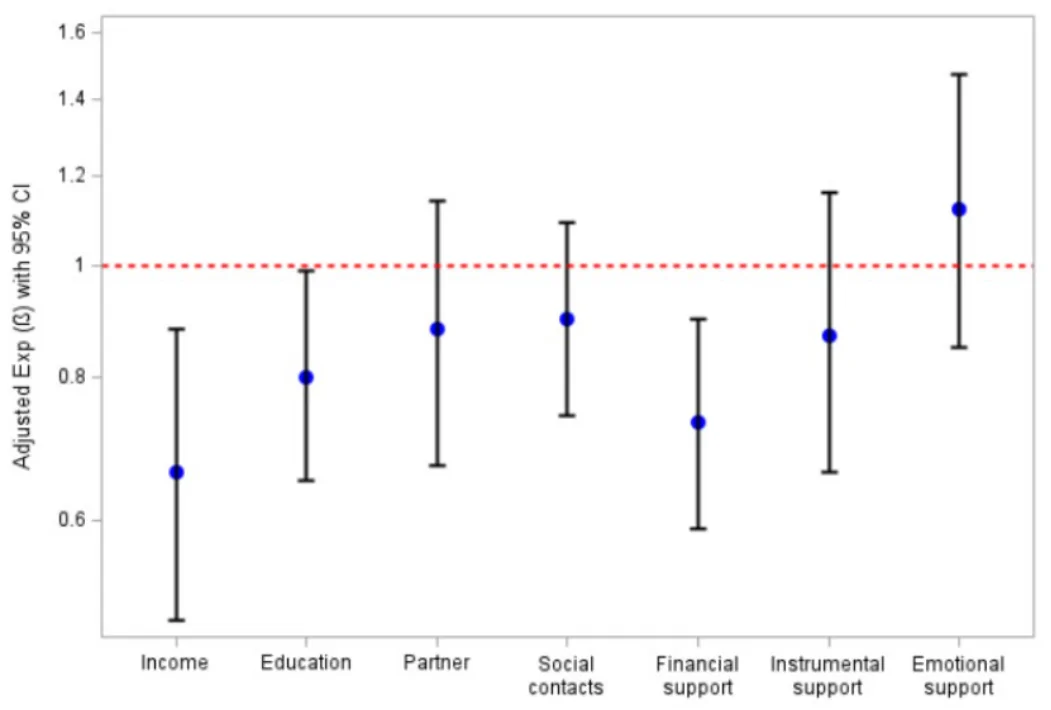
Associations between SES, social contacts and social support with incident diabetes: Results from AFT models ^a,b,c^. AFT: accelerated failure time; Q1: lowest quartile; Q4: highest quartile; a: For adjustment sets, see Methods section. b: Exposure contrasts: Income (low (Q1) versus high (Q4)), education (ISCED-97: up to higher secondary versus post-secondary), partner (no versus yes), social contacts (level 1 + 2 versus level 3 + 4 (many contacts)), financial, instrumental, emotional support (available, but insufficient/not available versus available and sufficient/available, but not needed). c: How to read: In participants with a household income in the lowest quartile, the expected time until diabetes is 0.66 times the expected time among participants with a household income in the highest quartile.

Adjusted AFT models indicate that the expected time to incident diabetes is shorter for individuals with lower SES than for individuals with higher SES (Figure 1). For example, for persons in the lowest income quartile, the expected time to incident diabetes is 0.66 (95% CI: 0.49–0.88) times as long as that of individuals in the highest income quartile.

Individuals with unavailable or insufficient financial support have an expected time to incident diabetes that is 0.73 (95% CI: 0.59–0.90) times as long as that of individuals who either received sufficient support or did not require it. For SII and social support, sex-stratified AFT models did not show any pronounced differences between men and women (Supplementary Table S4).

## 4. Discussion

In this study, associations between SES and incident type 2 diabetes were consistent with findings from previous research [16]. However, the present study does not confirm an association between limited social integration or the absence of a partnership and an increased risk of developing diabetes. An elevated diabetes risk was observed in individuals with limited financial support, but not in those with limited instrumental or emotional support. Financial support accounted for 14% of the effect of household income on diabetes. Only 2 to 5% of the effect of SES on diabetes was attributed to social integration and instrumental support. There was hardly any evidence of excess diabetes risk among individuals with both low education and low household income and poor social support.

Financial support and household income must be distinguished from each other, although these concepts are related to each other. Household income is an indicator of socioeconomic status (SES) referring to the financial resources available to a household to meet basic living needs, participate in socially expected activities, and signal social position. Financial support refers to additional resources available from relatives and friends that can buffer a lack of financial resources. It is not conceptualized as a core dimension of SES but is closely related to household income. In the present study, the proportion of individuals reporting low financial support was higher among those with household income below the median, whereas the proportion of individuals reporting available but unneeded financial support increased with higher household income.

In earlier studies, robust associations were mainly found for social isolation and loneliness. Several mechanisms have been proposed to explain the increased diabetes risk in individuals with limited social integration [24]. For instance, social integration may buffer stress, which is itself a known risk factor for diabetes [25]. It may also facilitate the spread of health-related behaviors, both protective and harmful, as has been demonstrated in the case of obesity [26]. Nevertheless, findings regarding the relationship between social integration and incident diabetes remain inconsistent. A meta-analysis suggests that social isolation is a significant risk factor for diabetes (pooled HR = 1.20; 95% CI: 1.10–1.43) [4], possibly because social isolation represents a particularly severe form of limited integration. In contrast, findings for other operationalizations of limited social integration are inconclusive [9,10,11,14]. For example, the same validated instrument for assessing the availability of social integration (AVSI) was used in two different studies [10,12]. In one study, no effect was observed in men (HR = 0.9; 95% CI: 0.5–1.7) [10], whereas the other found a statistically significant effect (HR = 1.93; 95% CI: 1.03–3.60) [12].

The finding that a lack of financial support from friends and relatives was independently associated with incident diabetes is novel. Limited financial support may increase stress, which is an established risk factor for diabetes [25]. Furthermore, a lack of financial support can reduce access to healthy food, fitness facilities and medical care. Only a few earlier studies suggest that social support may reduce the risk of developing diabetes [10,11,14,15]. In one such study, an age- and sex-adjusted hazard ratio of 0.98 (95% CI: 0.97–0.99) was reported for each additional point on a 0–36 social support scale [11]. Two other studies found a risk reduction in women with limited emotional and general social support [10,14]. In two US cohort studies, consistent associations were found for loneliness, not being married and spousal strain, but not for spousal or family social support [27].

It is noteworthy that in many studies examining the association between social integration or social support and incident diabetes, potential mediators such as sleep disorders, depression, and lifestyle factors were adjusted for [9,11,12,13]. Hendryx et al. reported that up to 37% of the associations between social support or social strain and diabetes could be explained by individual mediators, including BMI, depression, physical activity, diet, smoking, and alcohol consumption [14]. Holt-Lunstad et al. emphasized that associations between social relationships and mortality were stronger when more comprehensive instruments were used to assess social exposures [2]. This suggests that a further reason for the inconsistent findings across studies may lie in the variability of instruments used to measure social networks and social support.

In the present study, an indirect effect of 14% was found for the association between household income and incident diabetes via financial support. However, the confidence interval was wide, ranging from −8% to +38%. As mentioned below under limitations, some assumptions for mediator analyses might have been violated. Nevertheless, such an indirect effect seems plausible because poor households received less financial support from their social networks than better-off households, and because a lack of financial support increased the risk of diabetes. Nevertheless, the association between income and financial support was only moderate. The lack of financial support differed only slightly between people with household incomes below and above the median (23% versus 19%). Thus, income level alone predicts lack of financial support only to a small extent, so that financial support is a proxy for financial strain hardly captured by income quartiles. Only small indirect effects were observed for lack of social integration and lack of instrumental support as mediators. This is consistent with previous findings from the HNR Study, where similar small reductions were observed for subjective health as the outcome variable [28]. However, the results of the earlier study on mediation did not rely on a causal mediation analysis but rather on the percentage change in the effect estimates after potential mediators were added to the regression models.

In the current analysis, barely any interactions were seen between education and household income and dimensions of social support. It is possible that the study lacked statistical power to detect interaction effects between low social support/low social integration and low income/low social support with regard to diabetes incidence.

An earlier HNR-based study reported “partial support” for an interaction in analyses focusing on subjective health and depression [29]. A recent scoping review found that in 15 out of 25 studies, social network variables showed either mediating effects or interaction across various health outcomes [17].

### Strengths and Limitations

A strength of this study is its large sample size of 3642 participants and the long follow-up period of approximately 10 years. In addition, four distinct dimensions of social relationships were assessed (network size, instrumental support, financial support, and emotional support), and diabetes was defined using four different diagnostic criteria. Unlike some previous studies, we carefully avoided adjusting for potential mediators. The study also has limitations. First, some assumptions for mediator analyses may not be fulfilled in our study. The exposure and the mediator variables were measured at baseline so that it cannot be taken for granted that the mediator acts after the exposure. Moreover, there are three assumptions for mediation analyses that may not be fulfilled. These assumptions include control of exposure–mediator and of mediator–outcome confounding, and, furthermore, that the exposure does not affect any of the mediator–outcome confounders [30]. Second, it was not possible to include more than one mediator variable at a time in the causal mediation analyses in SAS, which may have resulted in larger mediation effects. Third, at the follow-up assessment (T1), social support was not measured using the same instruments as at baseline, which precluded the use of time-dependent exposure models for these variables. Forth, measurements on social support generally rely on self-reports and may be more subjective than information on social networks. Fifth, oral glucose tolerance tests were not conducted, so individuals with impaired glucose tolerance could not be identified in the diabetes case definition. Sixth, the HNR Study is an urban German cohort recruited in 2000–2003, and data of a 10-year follow-up were used. Financial support by relatives and friends may also depend on the welfare state, on cultural contexts (individualistic versus collectivistic societies) and intergenerational differences. Therefore, generalizability of the results to later times or other populations may not be taken for granted.

## 5. Conclusions

This study found a moderate effect of a lack of financial support on incident diabetes. As the present study is explorative, this finding needs replication in further studies. Furthermore, financial support may play a small mediating role in the well-established association between social inequality and diabetes risk. Again, this result of our mediation analysis needs cautious interpretation for reasons given under limitations.

In epidemiological research, no single study can offer definitive answers; rather, each contributes to the broader understanding of complex health relationships [31]. Given the limited body of literature, further studies on social relationships and diabetes are needed—ideally with greater methodological comparability. This includes the use of standardized instruments for measuring social relationships, consistent exposure contrasts, and the avoidance of over-adjustment for mediators.

## Figures and Tables

**Table 1:** Baseline characteristics of the study population (T0): the Heinz Nixdorf Recall Study.

N		3642
Variables (Missing)		
Age	Mean ± SD	58.9 ± 7.6
Sex	Male (n, (%))	1746 (47.9)
ISCED-97 (5)	1 (primary education)	40 (1.1)
	2 (lower secondary education)	319 (8.8)
	3 (upper secondary education)	2028 (55.8)
	4 (post-secondary non tertiary education)	107 (2.9)
	5 (tertiary education)	1143 (31.4)
Household income (€) (229)	Median (q1, q3)	1448 (1107, 1917)
Partner	No (n, (%))	553 (15.2)
SII (62)	Level I (isolation) (n, (%))	242 (6.8)
	Level II	1435 (40.1)
	Level III	1708 (47.7)
	Level IV (large network)	195 (5.5)
Financial support (12)	Available, and sufficient (n, (%))	522 (14.4)
	Available, but not needed	2348 (64.7)
	Available, but insufficient	37 (1.0)
	Not available	723 (19.9)
Instrumental support (64)	Available, and sufficient (n, (%))	2043 (57.1)
	Available, but not needed	1109 (31.0)
	Available, but insufficient	153 (4.3)
	Not available	273 (7.6)
Emotional support (15)	Available, and sufficient (n, (%))	2667 (73.5)
	Available, but not needed	395 (10.9)
	Available, but insufficient	283 (7.8)
	Not available	282 (7.8)
HbA1c [%]	Mean ± SD	5.3 ± 0.4

SII: social integration index.

**Table 2:** Associations between measures of socioeconomic status, social integration and social support with incident diabetes—results from a log-linear model with a Poisson working likelihood and robust standard errors.

Exposure	Exposure Categories	n	Number of Incident Cases of Diabetes (n, (%))	RR (95% CI)	
				Crude	Adjusted ^a^
Household income	Low (Q1) (≤1107 €)	770	109 (14.2)	1.17 (0.96–1.43)	1.18 (0.96–1.45)
	High (Q2–Q4) (>1107 €)	2643	320 (12.1)	1	1
Household income	Low (Q1) (≤1107 €)	770	109 (14.2)	1.39 (1.07–1.83)	1.38 (1.05–1.81)
	High (Q4) (>1917 €)	808	82 (10.2)	1	1
ISCED-97	Up to higher secondary	2387	306 (12.8)	1.12 (0.93–1.35)	1.19 (0.98–1.43)
	Post-secondary	1250	143 (11.4)	1	1
SII	Level I + II	1677	216 (12.9)	1.07 (0.90–1.27)	1.05 (0.87–1.26)
	Level III + IV (many contacts)	1903	229 (12.0)	1	1
Partner	No	553	72 (13.0)	1.07 (0.84–1.35)	1.09 (0.86–1.39)
	Yes	3086	377 (12.2)	1	1
Financial support	Available, but insufficient/not available	760	119 (15.7)	1.36 (1.12–1.65)	1.33 (1.09–1.62)
	Available, and sufficient/available, but not needed	2870	330 (11.5)	1	1
Instrumental support	Available, but insufficient/not available	426	61 (14.3)	1.18 (0.91–1.51)	1.12 (0.86–1.45)
	Available, and sufficient/available, but not needed	3152	384 (12.2)	1	1
Emotional support	Available, but insufficient/not available	565	66 (11.7)	0.93 (0.73–1.19)	0.89 (0.69–1.14)
	Available, and sufficient/available, but not needed	3062	383 (12.5)	1	1

RR: relative risk; CI: confidence interval; Q1: lowest quartile; Q4: highest quartile; ISCED: International Standard Classification of Education; SII: social integration index. ^a^ For adjustment sets, see Methods section.

**Table 3:** Results of mediation analyses—percentage mediated by various mediators for age–sex adjusted associations between household income/education and incident diabetes.

	Exposure	
Mediator	Household Income ^a,b^	Education ^c,d^
**SII ^e^**		
Total effect (RR, 95% CI)	1.176 (0.935, 1.417) ^g^	1.184 (0.953, 1.415)
Additional RR (95% CI) due to direct effect	0.168 (−0.072, 0.409) ^g^	0.177 (−0.053, 0.408)
Additional RR (95% CI) due to indirect effect	0.008 (−0.015, 0.031) ^g^	0.007 (−0.012, 0.025)
Percentage mediated (%, 95% CI)	4.4 (−9.7, 18.6) ^g^	3.6 (−7.1, 14.3)
**Financial support** ^f^		
Total effect (RR, 95% CI)	1.171 (0.932, 1.411)	1.187 (0.955, 1.419)
Additional RR (95% CI) due to direct effect	0.147 (−0.088, 0.383)	0.190 (−0.042, 0.422)
Additional RR (95% CI) due to indirect effect	0.024 (0.002, 0.047)	−0.003 (−0.014, 0.008)
Percentage mediated (%, 95% CI)	14.2 (−6.7, 35.0)	−1.6 (−7.5, 4.3)
**Emotional support** ^f^		
Total effect (RR, 95% CI)	1.177 (0.936, 1.419)	1.185 (0.954, 1.417)
Additional RR (95% CI) due to direct effect	0.183 (−0.060, 0.426)	0.187 (−0.045, 0.419)
Additional RR (95% CI) due to indirect effect	−0.005 (−0.021, 0.011)	−0.002 (−0.008, 0.005)
Percentage mediated (%, 95% CI)	−3.0 (−12.7, 6.6)	−0.8 (−4.7, 3.0)
**Instrumental support** ^f^		
Total effect (RR, 95% CI)	1.176 (0.936, 1.417)	1.184 (0.953, 1.415)
Additional RR (95% CI) due to direct effect	0.168 (−0.072, 0.408)	0.180 (−0.050, 0.410)
Additional RR (95% CI) due to indirect effect	0.009 (−0.009, 0.027)	0.004 (−0.004, 0.012)
Percentage mediated (%, 95% CI)	4.9 (−6.7, 16.5)	2.2 (−2.7, 7.1)

RR: relative risk, CI: confidence interval; SII: social integration index; ^a^ Q1 (lowest quartile, ≤1107€) versus Q2–Q4 (>1107 €); ^b^ N = 3324; ^c^ Up to higher secondary versus post-secondary; ^d^ N = 3533; ^e^ Level I/II (small network) versus Level III/IV (large network); ^f^ available, but insufficient/not available versus available and sufficient/available, but not needed. ^g^ How to read: Persons with a household income in the lowest quartile have a 1.176 times higher risk of diabetes than persons with a household income in the three higher quartiles. This relative risk increase of 17.6% can be divided into a relative risk increase of 16.8% due to the direct effect, and a relative risk increase of 0.8% due to the indirect effect. The percentage mediated is 0.008/0.176 × 100 = 4.4%.

**Table 4:** Analyses of interaction between education and social integration/social support with incident diabetes as the outcome.

				RR (95% CI)		
Education	SII	N	Cases of Incident T2DM	Model 1 (Crude)	Model 2 (Age–Sex Adjusted)	RERI (95% CI)
Low (ISCED-97 = “0”–“3”)	Poor (Level I + II)	1199	161 (13.4)	1.18 (0.92, 1.51)	1.26 (0.98, 1.62)	0.02 (−0.36, 0.40)
	Good (Level III + IV)	1147	143 (12.5)	1.09 (0.85, 1.41)	1.19 (0.93, 1.53)	
High (ISCED-97 = “4”, “5”)	Poor (Level I + II)	478	55 (11.5)	1.01 (0.73, 1.39)	1.05 (0.76, 1.44)	
	Good (Level III + IV)	755	86 (11.4)	1	1	
**Education**	**Financial support ^a^**					
Low (ISCED-97 = “0”–“3”)	Low	478	79 (16.5)	1.55 (1.18–2.03)	1.56 (1.19–2.04)	0.13 (−0.35, 0.62)
	High	1903	227 (11.9)	1.12 (0.90–1.39)	1.17 (0.94–1.46)	
High (ISCED-97 = “4”, “5”)	Low	282	40 (14.2)	1.33 (0.95–1.87)	1.25 (0.89–1.76)	
	High	965	103 (10.7)	1	1	
**Education**	**Instrumental support ^a^**					
Low (ISCED-97 = “0”–“3”)	Low	303	47 (15.5)	1.33 (0.98–1.81)	1.41 (1.03–1.93)	0.23 (−0.41, 0.86)
	High	2042	255 (12.5)	1.07 (0.88–1.31)	1.15 (0.94–1.40)	
High (ISCED-97 = “4”, “5”)	Low	123	14 (11.4)	0.98 (0.58–1.64)	1.04 (0.62–1.74)	
	High	1108	129 (11.6)	1	1	
**Education**	**Emotional support ^a^**					
Low (ISCED-97 = “0”–“3”)	Low	392	51 (13.0)	1.09 (0.81–1.48)	1.15 (0.85–1.56)	0.27 (−0.20, 0.74)
	High	1987	255 (12.8)	1.08 (0.88–1.31)	1.14 (0.94–1.40)	
High (ISCED-97 = “4”, “5”)	Low	173	15 (8.7)	0.73 (0.44–1.21)	0.74 (0.44–1.22)	
	High	1073	128 (11.9)	1	1	

RR: relative risk, CI: confidence interval, RERI: relative excess risk due to interaction, SII: social integration index. ^a^ “Low” support means “Available, but insufficient/not available”; “high” support means “Available, and sufficient/available, but not needed.”

## Data Availability

Due to data security reasons (i.e., data contain potential participant-identifying information), the HNR Study does not allow sharing data as a public use file. Data requests can be addressed to recall@uk-essen.de.

## Supplementary Materials

The following supporting information can be downloaded at https://www.mdpi.com/article/10.3390/epidemiologia7040106/s1, Table S1: Baseline characteristics of excluded participants: the Heinz Nixdorf Recall Study. Table S2: Associations between measures of social support and social integration with incident diabetes—results from a log-linear model with a Poisson working likelihood and robust standard errors. Table S3: Analyses of interaction between household income and social integration/social support with incident diabetes as the outcome. Table S4: Associations between measures of social contact and social support with incident diabetes—results from AFT models.

## Abbreviations

The following abbreviations are used in this manuscript:

HNRS	Heinz Nixdorf Recall Study
RERI	Relative Excess Risk due to Interaction
AFT	Accelerated Failure Time
RR	Relative Risk
CI	Confidence Interval
ISCED	International Standard Classification of Education
SES	Socioeconomic Status
SII	Social Integration Index
T2DM	Type 2 Diabetes
