# Supplementary material for: Social Integration, Social Support and Incident Type 2 Diabetes: Results from the Prospective Heinz Nixdorf Recall Cohort Study
Source: Epidemiologia (Basel). 2026 Aug 4;7(4):106. doi: 10.3390/epidemiologia7040106 (PMC13512196; doi:10.3390/epidemiologia7040106)
Supplement: Supplementary file 1 [file epidemiologia-07-00106-s001.zip › R1 Table S1.pdf]

Table S1. Baseline characteristics of excluded participants: the Heinz Nixdorf Recall Study

		**Diabetes or missing diabetes measurement at baseline (T0)**	**No participation at follow-up (T1 / T2) or missing diabetes measurement at follow-up**
**n**		623 [a]	549 [b]
Age	Mean ± SD	62.1 ± 7.5	61.4 ± 8.4
Sex	Male (n, (%))	375 (60.2)	274 (50.0)
ISCED-97	1 (primary education)	5 (0.8%)	5 (0.9%)
	2 (lower secondary education)	87 (14.0%)	91 (16.8)
	3 (upper secondary education)	341 (55.0%)	307 (56.8)
	4 (post-secondary non tertiary education)	14 (2.3%)	12 (2.2)
	5 (tertiary education)	173 (27.9%)	126 (23.3)
Household income (€)	Median (q1, q3)	1406 (977; 1875)	1278 (937; 1662)
Social integration index	Level I (isolation) (n, (%))	53 (8.7)	80 (15.2)
	Level II	272 (44.4)	262 (49.7)
	Level III	268 (43.8)	170 (32.3)
	Level IV (large network)	19 (3.1)	15 (2.9)
Financial support	Available, and sufficient (n, (%))	90 (14.5)	81 (15.0)
	Available, but not needed	380 (61.4)	308 (57.1)
	Available, but insufficient	10 (1.6)	8 (1.5)
	Not available	139 (22.5)	142 (26.4)
Instrumental support	Available, and sufficient (n, (%))	370 (60.6)	304 (57.8)
	Available, but not needed	169 (27.7)	126 (24.0)
	Available, but insufficient	22 (3.6)	43 (8.2)
	Not available	50 (8.2)	53 (10.1)
Emotional support	Available, and sufficient (n, (%))	430 (69.7)	377 (70.1)
	Available, but not needed	78 (12.6)	54 (10.0)
	Available, but insufficient	36 (5.8)	44 (8.2)
	Not available	73 (11.8)	63 (11.7)
HbA1c [%]	Mean ± SD	7.1 ± 1.4	5.4 ± 0.5

[a] 576 had diabetes at baseline. For another 47, there was no diabetes measurement at baseline.

[b] 523 did not participate in T1 and T2. For another 26, there was no diabetes measurement at follow-up.
