# Supplementary material for: Social Integration, Social Support and Incident Type 2 Diabetes: Results from the Prospective Heinz Nixdorf Recall Cohort Study
Source: Epidemiologia (Basel). 2026 Aug 4;7(4):106. doi: 10.3390/epidemiologia7040106 (PMC13512196; doi:10.3390/epidemiologia7040106)
Supplement: Supplementary file 1 [file epidemiologia-07-00106-s001.zip › R1 Table S2.pdf]

Table S2. Associations between measures of social support and social integration with incident diabetes – results from a log-linear model with a Poisson working likelihood and robust standard errors [a]

	n (%)	N	RR (crude) (95% CI)	RR (adjusted) (95% CI)
**Financial support**				
Not available	117 (16.2%)	723	1.44 (1.18 – 1.76)	1.41 (1.14 – 1.73)
Available, but insufficient	2 (5.4%)	37	0.48 (0.12 – 1.86)	0.57 (0.15 – 2.16)
Available and sufficient	66 (12.6%)	522	1.12 (0.87 – 1.45)	1.17 (0.90 – 1.52)
Available, not needed	264 (11.2%)	2348	1	1
**Instrumental support**				
Not available	49 (18.0%)	273	1.81 (1.32 – 2.47)	1.72 (1.24 – 2.37)
Available, but insufficient	12 (7.8%)	153	0.79 (0.45 – 1.40)	0.86 (0.48 – 1.57)
Available and sufficient	274 (13.4%)	2043	1.35 (1.10 – 1.67)	1.43 (1.16 – 1.78)
Available, not needed	110 (9.9%)	1109	1	1
**Emotional support**				
Not available	46 (16.3%)	282	1.29 (0.89 – 1.87)	1.28 (0.87 – 1.90)
Available, but insufficient	20 (7.1%)	283	0.56 (0.34 – 0.92)	0.65 (0.39 – 1.09)
Available and sufficient	333 (12.5%)	2667	0.99 (0.75 – 1.30)	1.13 (0.84 – 1.52)
Available, not needed	50 (12.7%)	395	1	1
**SII**				
Level I (isolated)	37 (15.3%)	242	1.10 (0.70 – 1.75)	1.10 (0.69 – 1.75)
Level II	179 (12.5%)	1435	0.90 (0.61 – 1.31)	0.89 (0.61 – 1.31)
Level III	202 (11.8%)	1708	0.85 (0.59 – 1.24)	0.86 (0.59 – 1.27)
Level IV (large network)	27 (13.9%)	195	1	1

RR: relative risk; CI: confidence interval; SII: social integration index

[a] Contrary to table 2 in the main text, the measures of social support and the SII were not dichotomized.
