# Supplementary material for: Social Integration, Social Support and Incident Type 2 Diabetes: Results from the Prospective Heinz Nixdorf Recall Cohort Study
Source: Epidemiologia (Basel). 2026 Aug 4;7(4):106. doi: 10.3390/epidemiologia7040106 (PMC13512196; doi:10.3390/epidemiologia7040106)
Supplement: Supplementary file 1 [file epidemiologia-07-00106-s001.zip › R1 Table S3.pdf]

**Table S3.** Analyses of interaction between household income and social integration / social support with incident diabetes as the outcome

| | | | | RR (95% CI) | | |
|---|---|---|---|---|---|---|
| **Household income** | **SII** | **N** | **Cases of incident T2DM** | **Model 1 (crude)** | **Model 2 (age-sex adjusted)** | **RERI (95% CI)** |
| Q1 (≤ 1107€) | Poor (Level I + II) | 426 | 58 (13.6) | 1.17 (0.89, 1.55) | 1.17 (0.89, 1.56) | -0.27 (-0.76, 0.23) |
| | Good (Level III + IV) | 327 | 49 (15.0) | 1.29 (0.96, 1.73) | 1.33 (0.99, 1.78) | |
| Q2 – Q4 (> 1107€) | Poor (Level I + II) | 1155 | 149 (12.9) | 1.11 (0.90, 1.37) | 1.11 (0.90, 1.37) | |
| | Good (Level III + IV) | 1455 | 169 (11.6) | 1 | 1 | |
| **Household income** | **Financial support** [a] | | | | | |
| Q1 (≤ 1107€) | Low | 192 | 30 (15.6%) | 1.42 (1.00, 2.01) | 1.37 (0.97, 1.94) | -0.33 (-0.94, 0.27) |
| | High | 574 | 79 (13.8) | 1.25 (0.98, 1.58) | 1.26 (0.99, 1.60) | |
| Q2 – Q4 (> 1107€) | Low | 512 | 85 (16.6) | 1.50 (1.20, 1.89) | 1.44 (1.15, 1.81) | |
| | High | 2130 | 235 (11.0) | 1 | 1 | |
| **Household income** | **Instrumental support** [a] | | | | | |
| Q1 (≤ 1107€) | Low | 122 | 20 (16.4) | 1.36 (0.90, 2.06) | 1.37 (0.90, 2.07) | 0.11 (-0.58, 0.79) |
| | High | 632 | 87 (13.8) | 1.14 (0.91, 1.43) | 1.16 (0.92, 1.45) | |
| Q2 – Q4 (> 1107€) | Low | 273 | 36 (13.2) | 1.09 (0.79, 1.51) | 1.11 (0.80, 1.53) | |
| | High | 2335 | 282 (12.1) | 1 | 1 | |
| **Household income** | **Emotional support** [a] | | | | | |
| Q1 (≤ 1107€) | Low | 154 | 22 (14.3) | 1.15 (0.77, 1.73) | 1.14 (0.76, 1.70) | 0.11 (-0.47, 0.68) |
| | High | 611 | 87 (14.2) | 1.15 (0.92, 1.44) | 1.17 (0.94, 1.46) | |
| Q2 – Q4 (> 1107€) | Low | 377 | 40 (10.6) | 0.86 (0.63, 1.17) | 0.86 (0.63, 1.18) | |
| | High | 2263 | 280 (12.4) | 1 | 1 | |

RR: relative risk, CI: confidence interval, RERI: relative excess risk due to interaction, SII: social integration index

[a] "Low" support means "Available, but insufficient / not available", "high" means "Available, and sufficient / available, but not needed"
