# Supplementary material for: Social Integration, Social Support and Incident Type 2 Diabetes: Results from the Prospective Heinz Nixdorf Recall Cohort Study
Source: Epidemiologia (Basel). 2026 Aug 4;7(4):106. doi: 10.3390/epidemiologia7040106 (PMC13512196; doi:10.3390/epidemiologia7040106)
Supplement: Supplementary file 1 [file epidemiologia-07-00106-s001.zip › R1 Table S4.pdf]

Table S4. Associations between measures of social contact and social support with incident diabetes – results from AFT models

Exposure	Exposure categories	n	Number of incident cases of diabetes (n, (%))	Adjusted Exp (ß) from AFT models (95% CI) [a,b]		
				Total	men	women
Social contacts	Level 1 + 2	1677	216 (12.9)	0.90 (0.74, 1.09)	0.78 (0.60, 1.01)	1.03 (0.77, 1.38)
	Level 3 + 4 (many contacts)	1903	229 (12.0)	1	1	1
Partner	No	553	72 (13.0)	0.88 (0.67 – 1.14)	0.73 (0.45, 1.19)	0.93 (0.68, 1.28)
	Yes	3086	377 (12.2)	1	1	1
Financial support	Available, but insufficient / not available	760	119 (15.7)	0.73 (0.59, 0.90)	0.79 (0.59, 1.05)	0.67 (0.48, 0.93)
	Available, and sufficient / available, but not needed	2870	330 (11.5)	1	1	1
Instrumental support	Available, but insufficient / not available	426	61 (14.3)	0.87 (0.66, 1.16)	0.74 (0.50, 1.08)	1.05 (0.69, 1.61)
	Available, and sufficient / available, but not needed	3152	384 (12.2)	1	1	1
Emotional support	Available, but insufficient / not available	565	66 (11.7)	1.12 (0.85, 1.47)	1.21 (0.83, 1.79)	1.06 (0.72, 1.55)
	Available, and sufficient / available, but not needed	3062	383 (12.5)	1	1	1

AFT: accelerated failure time, CI: confidence interval; Q1: lowest quartile; Q4: highest quartile; ISCED: International Standard Classification of Education

[a] For adjustment sets, see methods section

[b] **How to read**: In men without a partner, the expected time until diabetes is 0.73 times the expected time among men with a partner.
